# A survey of RNA viruses in mosquitoes from Mozambique reveals novel genetic lineages of flaviviruses and phenuiviruses, as well as frequent flavivirus-like viral DNA forms in *Mansonia*

**DOI:** 10.1186/s12866-020-01905-5

**Published:** 2020-07-28

**Authors:** Ana Paula Abílio, Manuel Silva, Ayubo Kampango, Inácio Narciso, Eduardo Samo Gudo, Luís Carlos Bernardo das Neves, Mohsin Sidat, José Manuel Fafetine, António Paulo Gouveia de Almeida, Ricardo Parreira

**Affiliations:** 1 Instituto Nacional de Saúde (INS)-Ministry of Health (MISAU), Vila de Marracuene, Estrada Nacional N°1, Parcela N°3943, P.O. Box: 264, Maputo, Mozambique; 2 Faculty of Medicine, Eduardo Mondlane University (UEM), Maputo, Mozambique; 3 Unidade de Microbiologia Médica, Instituto de Higiene e Medicina Tropical (IHMT)/Universidade Nova de Lisboa (NOVA), and Global Health and Tropical Medicine (GHTM) Research Centre, Lisbon, Portugal; 4 Unidade de Parasitologia Médica, Instituto de Higiene e Medicina Tropical (IHMT)/Universidade Nova de Lisboa (NOVA), and Global Health and Tropical Medicine (GHTM) Research Centre, Lisbon, Portugal; 5 Faculty of Veterinary Sciences Pretoria, University of Pretoria (UP), Pretoria, South Africa; 6 Faculty of Veterinary, Eduardo Mondlane University (UEM), Maputo, Mozambique

**Keywords:** Flaviviruses, Bunyaviruses, Mosquitoes, Viral DNA forms, Phylogenetic analysis, Mozambique

## Abstract

**Background:**

Mosquito-borne diseases involving arboviruses represent expanding threats to sub-Saharan Africa imposing as considerable burden to human and veterinary public health. In Mozambique over one hundred species of potential arbovirus mosquito vectors have been identified, although their precise role in maintaining such viruses in circulation in the country remains to be elucidated. The aim of this study was to screen for the presence of flaviviruses, alphaviruses and bunyaviruses in mosquitoes from different regions of Mozambique.

**Results:**

Our survey analyzed 14,519 mosquitoes, and the results obtained revealed genetically distinct insect-specific flaviviruses, detected in multiple species of mosquitoes from different genera. In addition, smaller flavivirus-like *NS5* sequences, frequently detected in *Mansonia* seemed to correspond to defective viral sequences, present as viral DNA forms. Furthermore, three lineages of putative members of the *Phenuiviridae* family were also detected, two of which apparently corresponding to novel viral genetic lineages.

**Conclusion:**

This study reports for the first-time novel insect-specific flaviviruses and novel phenuiviruses, as well as frequent flavivirus-like viral DNA forms in several widely known vector species. This unique work represents recent investigation of virus screening conducted in mosquitoes from Mozambique and an important contribution to inform the establishment of a vector control program for arbovirus in the country and in the region.

## Background

Vector-borne diseases caused by arboviruses such as the Rift Valley fever, dengue, chikungunya, Zika, or West Nile viruses (RVFV, CHIKV, DENV, ZIKV and WNV, respectively), represent emerging and expanding threats in sub-Saharan Africa, and remain a major burden to global health, despite increasing funding allocated for their control and eradication [1]. Every year, more than one billion humans are infected, many of who die from vector-borne viral diseases, and more than half of the world’s population may currently be at risk of infection, particularly in low-income countries [2, 3].

Our knowledge of the diversity of the viral world has significantly expanded over the last decade. During this period, a large number of studies have shown that viruses are the most abundant biological entities on the planet and display a remarkable degree of genetic diversity and genomic plasticity [4, 5], and have also allowed us to bridge apparent phylogenetic gaps in the virosphere. This is especially true when viral surveys focus on rarely sampled *taxa* or infrequently visited biotopes, and revealing novel or divergent viral groups [6–10].

Invertebrates are among the animals most frequently sampled in recent viral surveys, and their viromes seem to include a large number of genetically diverse viruses [9]. Mosquitoes (Diptera: Culicidae) are clearly the invertebrates most commonly studied due to their role as vectors of pathogenic viruses to humans and other animals [11]. However, the viromes of mosquitoes have been shown not to be limited to the latter, many of which (e.g. dengue, yellow fever or Zika viruses) have become household names in recent times. In fact, mosquitoes also host a profusion of viruses that only infect invertebrate cells and are, therefore, regarded as insect-restricted [12–14]. On the other hand, viral surveys are still frequently carried out in association with disease outbreaks, or when identifiable factors increase the probability for an arbovirus to (re)emerge and/or rapidly disperse [11]. Moreover, since there is limited knowledge on the genetic diversity, and ecology, of viruses in their natural enzootic maintenance cycles, little is also known regarding the adaptive constraints ruling the evolutionary steps that determine arbovirus emergence from their sylvatic niches [15].

Mozambique is located in a region suitable to arbovirus outbreaks, and in recent times the country was affected by two dengue virus outbreaks, which occurred in the northern regions [16, 17]. Increasing evidence also suggest that the country may be endemic to other debilitating and life-threatening arboviral threats including RVFV [18–20], DENV [2, 16, 21] and CHIKV [22, 23]. Moreover, historical and global risk projection have suggested that the country may also be suitable for the establishment of ZIKV [24–26], a virus recently linked to cases of microcephaly as well as many other neurological abnormalities in newly born infants [27]. Despite increasing evidence indicating the circulation of public heath-relevant arboviruses in Mozambique, the burden of the diseases they cause remains unknown. In addition, more than a hundred potentials arbovirus vectors have been identified in Mozambique, and these include *Aedes spp*, *Culex spp*, *Mansonia spp* and *Anopheles spp* [28–31], of which their role in maintaining arboviruses in nature remain to be elucidated.

The focus of this study was the detection, and analysis, of selected *taxa* of RNA viruses in different geographic regions in Mozambique. These regions display rich mosquito and wildlife faunas, as well as bioecological features that allow mosquitoes, wildlife, domestic animals and humans to coexist in close proximity. The viruses targeted in this viral survey included alphaviruses, flaviviruses, and different bunyaviruses. While our initial interest as far as bunyaviruses were concerned involved the detection of RVFV, in a subset of samples the viral screening also included detection of phlebovirus-like and orthobunyavirus genomes. The results obtained did not reveal the circulation of recognizable pathogenic viruses in wild-caught mosquitoes, but uncovered divergent phenuiviruses, as well as different lineages of insect-specific flaviviruses.

## Results

**Fig. 1:**
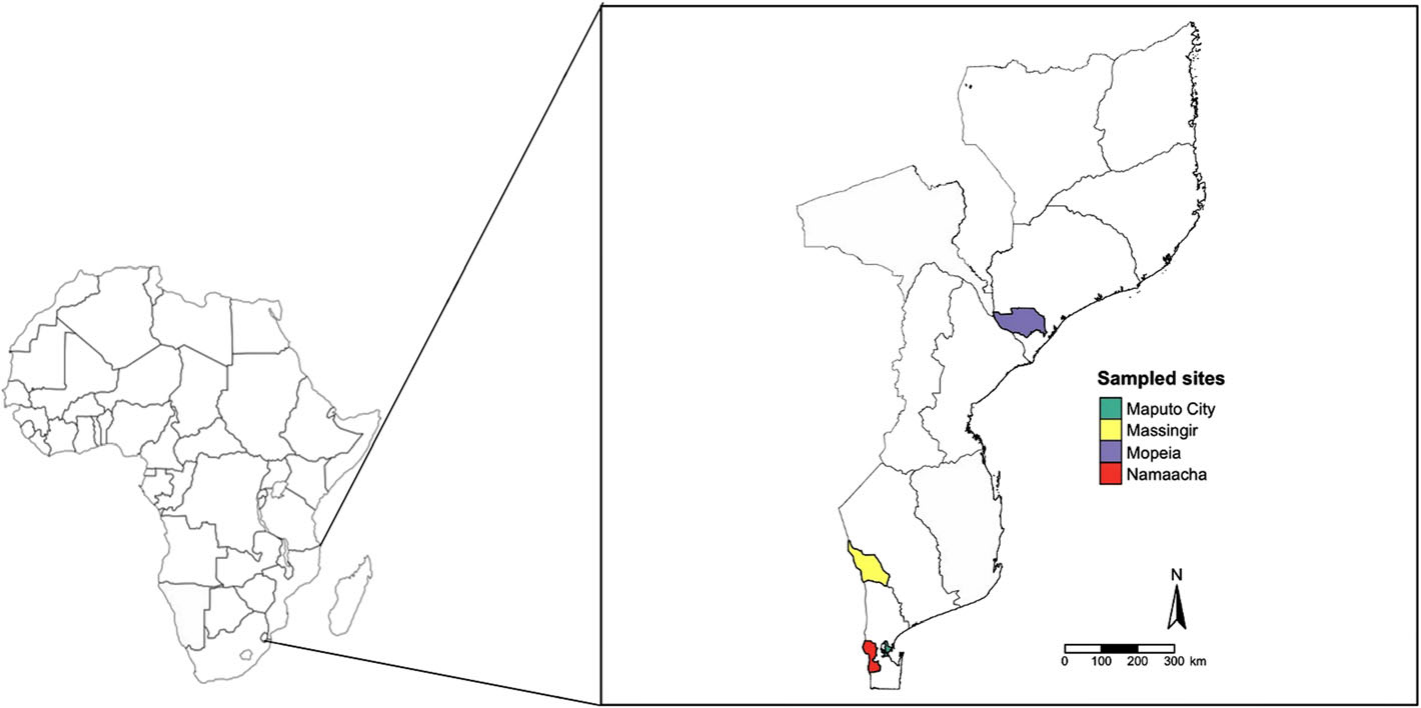
Geographic coverage in Mozambique (west southern Africa) of the mosquito collections described in this report. The provinces indicated and the different municipalities where mosquito collections were carried out are color-coded

The results presented in this work were based on the analysis of a total of 14,519 mosquitoes, collected in 3 regions of Mozambique (Fig. 1) during 12 successive collection campaigns, carried out between November/2014 and December/2015. The majority 45.55% (*n* = 6614/14519) of the screened mosquitoes were classified as *Culex spp.*, followed by *Anopheles spp*. 27.16% (*n* = 2943/14519) and *Mansonia spp* 25.22% (*n* = 3662/14519). Mosquitoes were grouped into 351 pools, ranging from 1 to a maximum of 128 specimens, with the average of (approximately) 41 mosquitoes each. These were subsequently processed by RT-PCR for the detection of specific viral agents (such as RVFV), or groups of viruses (such as alphaviruses and flaviviruses).

### Analysis of flavivirus sequences

The genomes of flaviviruses were targeted using the primers previously described by Vázquez et al., (2012), which reveal an amplicon with the expected mass of ≈1 kbp [that on the Culex flavivirus strain CxFV-Mex07 reference sequence (EU879060) would define a section of the viral genome from coordinates 9800 to 9901] in the cDNA extracts prepared from 45/351 pools (12.8%). These results indicated the presence of flavivirus genome in 9 different species of mosquitoes from possibly 4 genera (*Anopheles*, *Culex Coquillettidia,* and *Mansonia*). A sample (*n* = 20) of these amplicons was sequenced, and BLASTn/x similarity searches unambiguously confirmed they had a flavivirus origin. Similarly, the mosquito species of the pool of origin was confirmed by analysis of *COI* sequences in all but 5 pools, for lack of a PCR product. These corresponded to three of *Ma. (Mnd) africana*, and two of *Ma. (Mnd) uniformis*, all of which are very distinctive and clearly identifiable *taxa*. Table 1 lists all the viral sequences obtained in this study, as well as the date and location of collection of respective mosquito pools, their species, and respective accession numbers.

**Table 1:** Date of collection and location of positive *flavivirus* and *phenuivirus-*like sequence detection in mosquitoes from Mozambique, and their respective accession numbers

Date of collection	Locality/District	Province	Geo - references	Pool lab code (size)	Mosquito species^a^	Flavivirus sequence	Phenuivirus sequence	COX1 mosquito sequence
03/12/14	Chinuara/ Mopeia	Zambézia	17° 79′ 04. 748″ S; 035° 4′ 1442″ E	Moz 3 (*n* = 50)	*Mansonia africana*	LC462008	–	LC517270
04/12/14	Chinuara/ Mopeia	Zambézia	17° 79′ 04. 748″ S; 035° 4′ 1442″ E	Moz 9 (*n* = 55)	*Mansonia africana*	LC462001	–	LC517271
27/04/15	Chinuara/ Mopeia	Zambézia	17° 79′ 04. 748″ S; 035° 4′ 1442″ E	Moz 19 (*n* = 50)	*Mansonia uniformis*	LC462246	–	n.a.
04/06/15	Limpopo park/ Massingir	Gaza	23° 52′ 04.4399″ S; 032° 08′ 43.0852″ E	Moz 38 (*n* = 20)	*Anopheles* sp.	LC462010	–	LC517272
26/11/14	Limpopo park/ Massingir	Gaza	23° 52′ 04.4399″ S; 032° 08′ 43.0852″ E	Moz 39 (*n* = 14)	*Anopheles* sp.	LC462009	–	LC517273
18/11/14	Goba/Namaacha	Maputo	26° 3′ 59.73″ S; 032° 10′ 23.36″ E	Moz 47 (*n* = 48)	*Anopheles coustani*	–	LC461999	LC517274
19/12/14	Goba/Namaacha	Maputo	26° 3′ 59.73″ S; 032° 10′ 23.36″ E	Moz 54 (*n* = 39)	*Culex tritaeniorhynchus*	–	LC461994	LC517275
							LC461995	
28/01/15	Goba/Namaacha	Maputo	26° 3′ 59.73″ S; 032° 10′ 23.36″ E	Moz 76 (*n* = 40)	*Mansonia uniformis*	LC462253	–	LC517276
28/01/15	Goba/Namaacha	Maputo	26° 3′ 59.73″ S; 032° 10′ 23.36″ E	Moz 77 (*n* = 64)	*Mansonia africana*	LC462249	–	LC517277
27/04/15	Chinuara/ Mopeia	Zambézia	17° 79′ 04. 748″ S; 035° 4′ 1442″ E	Moz 89 (*n* = 50)	*Mansonia africana*	LC462005	–	LC517278
27/04/15	Chinuara/ Mopeia	Zambézia	17° 79′ 04. 748″ S; 035° 4′ 1442″ E	Moz 90 (n = 50)	*Mansonia uniformis*	LC462251	–	LC517279
01/10/15	Goba/Namaacha	Maputo	26° 3′ 59.73″ S; 032° 10′ 23.36″ E	Moz 96 (n = 50)	*Mansonia africana*	LC462016	–	LC517280
21/07/15	Goba/Namaacha	Maputo	26° 3′ 59.73″ S; 032° 10′ 23.36″ E	Moz 97 (*n* = 12)	*Anopheles coustani*	–	LC462000	LC517281
21/07/15	Goba/Namaacha	Maputo	26° 3′ 59.73″ S; 032° 10′ 23.36″ E	Moz 98 (*n* = 18)	*Anopheles coustani*	LC462013	–	LC517282
28/01/15	Goba/Namaacha	Maputo	26° 3′ 59.73″ S; 032° 10′ 23.36″ E	Moz 104 (*n* = 53)	*Mansonia uniformis*	LC462007	–	LC517283
27/04/15	Chinuara/ Mopeia	Zambézia	17° 79′ 04. 748″ S; 035° 4′ 1442″ E	Moz 160 (n = 50)	*Anopheles pretoriensis*	LC462011	LC461996	LC517284
							LC461997	
27/04/15	Chinuara/ Mopeia	Zambézia	17° 79′ 04. 748″ S; 035° 4′ 1442″ E	Moz 161 (*n* = 49)	*Anopheles pretoriensis*	LC462012	–	LC517285
09/01/15	Chinuara/ Mopeia	Zambézia	17° 79′ 04. 748″ S; 035° 4′ 1442″ E	Moz 166 (*n* = 26)	*Anopheles pretoriensis*	LC462015	LC461998	LC517286
09/01/15	Chinuara/ Mopeia	Zambézia	17° 79′ 04. 748″ S; 035° 4′ 1442″ E	Moz 182 (n = 50)	*Culex antennatus*	LC462017	–	LC517287
09/01/15	Chinuara/ Mopeia	Zambézia	17° 79′ 04. 748″ S; 035° 4′ 1442″ E	Moz 212 (n = 50)	*Culex antennatus*	LC462254	–	LC517288
						LC462255		
03/12/14	Chinuara/ Mopeia	Zambézia	17° 79′ 04. 748″ S; 035° 4′ 1442″ E	Moz 258 (n = 50)	*Mansonia africana*	LC462003	–	LC517289
03/12/14	Chinuara/ Mopeia	Zambézia	17° 79′ 04. 748″ S; 035° 4′ 1442″ E	Moz 259 (*n* = 54)	*Mansonia africana*	LC462002	–	n.a.
03/12/14	Chinuara/ Mopeia	Zambézia	17° 79′ 04. 748″ S; 035° 4′ 1442″ E	Moz 263 (*n* = 75)	*Mansonia africana*	LC462004	–	n.a.
03/12/14	Chinuara/ Mopeia	Zambézia	17° 79′ 04. 748″ S; 035° 4′ 1442″ E	Moz 269 (*n* = 51)	*Mansonia africana*	LC462006	–	n.a.
09/01/15	Chinuara/ Mopeia	Zambézia	17° 79′ 04. 748″ S; 035° 4′ 1442″ E	Moz 299 (*n* = 45)	*Mansonia uniformis*	LC462250	–	LC517290
						LC462256		
20/12/14	Goba/Namaacha	Maputo	26° 3′ 59.73″ S; 032° 10′ 23.36″ E	Moz 309 (*n* = 8)	*Mansonia uniformis*	LC462252	–	n.a.
30/07/15	Chinuara/ Mopeia	Zambézia	17° 79′ 04. 748″ S; 035° 4′ 1442″ E	Moz 313 (n = 50)	*Mansonia africana*	LC462247	–	LC517291
						LC462257		
30/07/15	Chinuara/ Mopeia	Zambézia	17° 79′ 04. 748″ S; 035° 4′ 1442″ E	Moz 319 (n = 50)	*Mansonia africana*	LC462014	–	LC517292
26/06/15	Chinuara/ Mopeia	Zambézia	17° 79′ 04. 748″ S; 035° 4′ 1442″ E	Moz 324 (n = 1)	*Mansonia uniformis*	LC462019	–	LC517293
03/12/14	Chinuara/ Mopeia	Zambézia	17° 79′ 04. 748″ S; 035° 4′ 1442″ E	Moz 341 (n = 5)	*Coquillettidia metallica*	LC462018	–	LC536568

^a^Identification to the species level was confirmed based on COX1 barcoding using sequences generated by Sanger (population) sequencing of PCR products, except where marked with “n.a.” in COX1 mosquito sequence accession numbers column

**Fig. 2:**
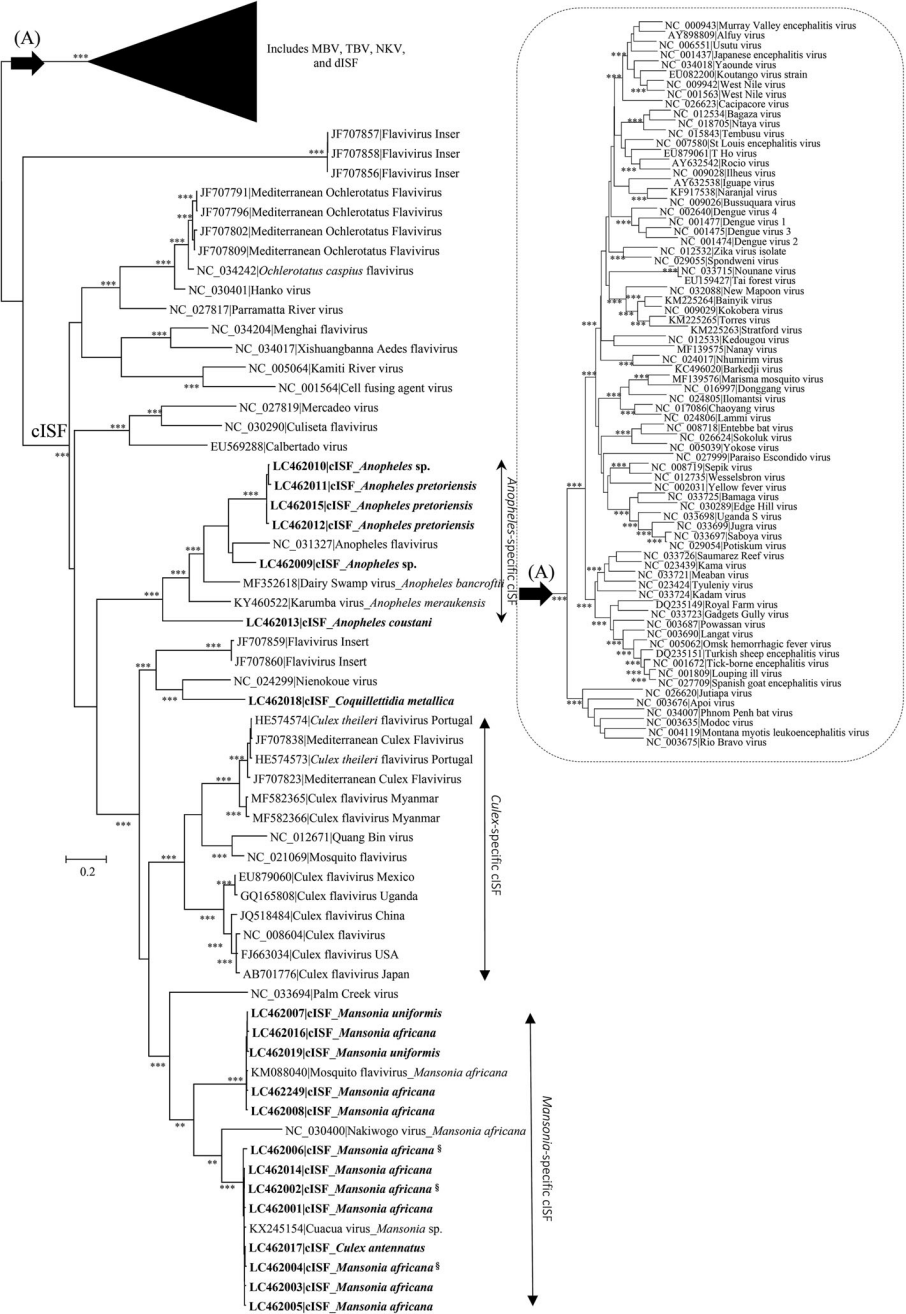
**a** Phylogenetic analysis of flavivirus *NS5* nucleotide sequences (≈1 kbp per sequence). At specific branches, the number of “*” indicates the branch-support as revealed by the different phylogenetic reconstructions methods used, and assuming as relevant bootstrap values ≥75% (using 1000 resamplings of the sequence data in maximum likelihood analysis) and posterior probability values ≥0.80 (when Bayesian approaches were used). One, two or three “*” would indicate that a given branch had been supported by one, two, or all the phylogenetic reconstruction approaches used in the amalysis (ML and Bayesian analysis using two sets of demographic priors). At the top of the tree, the collapsed monophyletic group including reference sequences from mosquito-borne viruses (MBV), tick-borne viruses (TBV), no known vector viruses (NKV), and dual-host associated insect-specific viruses (dISF), while the branches shown comprise the so-called classical insect-specific flaviviruses (cISF), is expanded at the right (**b**). The sequences described in this work are indicated in bold-face. All the sequences used are designated by their respective accession numbers|virus name. The size bar indicates the number of nucleotide substitutions per site. §-Mosquito species could not be confirmed by *COI* sequence

To further extend the characterization of the viral sequences obtained, a phylogenetic analysis was carried out using different methods. In all cases, the obtained phylogenetic trees indicated that none of the analyzed sequences had been amplified from bona fide arboviruses. Indeed, this is clearly revealed by their exclusion from the monophyletic cluster that assembles mosquito-borne and tick-borne flaviviruses in phylogenetic trees (cluster A in Fig. 2), the composition of which is shown in detail in the dotted box (indicated by the arrow). Conversely, all the sequences obtained in this study segregated within the large monophyletic group that assembles the so-called classical insect-specific flaviviruses, or cISF [12]. Furthermore, the analysis of the tree topologies obtained clearly suggested they did not group together in a single cluster, but rather segregated (i) either with other known viral sequences or (ii) formed independent genetic lineages. One of these lineages includes only sequences amplified from *Anopheles* spp. mosquitoes, while two others, also sharing a common ancestry, were mostly found in *Mansonia spp.* Unexpectedly, one of these sequences (LC462017) was obtained from a pool of mosquitoes identified as *Culex (Cux) antennatus* (pool Moz 182). However, the association of an apparently *Culex*-derived viral sequence with this group was considered debatable given its high similarity with the viral sequences amplified from *Mansonia* (see discussion). The above mentioned lineages of cISF include the Cuacua virus, previously identified in *Mansonia* sp. [29]. In this work, NS5-coding sequences 98–100% identical to those of the Cuacua virus were described both in *Ma. africana* and *Ma. uniformis*.

One of the other lineages of cISF identified is represented by a viral sequence obtained from *Cq. metallica* which clustered with that of Nienokoue virus (NC_024299) from *Culex* sp. However, these sequences share only 76.1% of sequence identity (as defined by Blast2 sequence comparison), clearly below the 84% limit defined by Kuno and others [32] and, therefore, indicating that they represent distinct viral species. The remainder flavivirus lineages were detected in pools of *Anopheles* mosquitoes, four of which could be classified to the species level as *An. (Cel) pretoriensis* and *An. (Ano) coustani*.

**Fig. 3:**
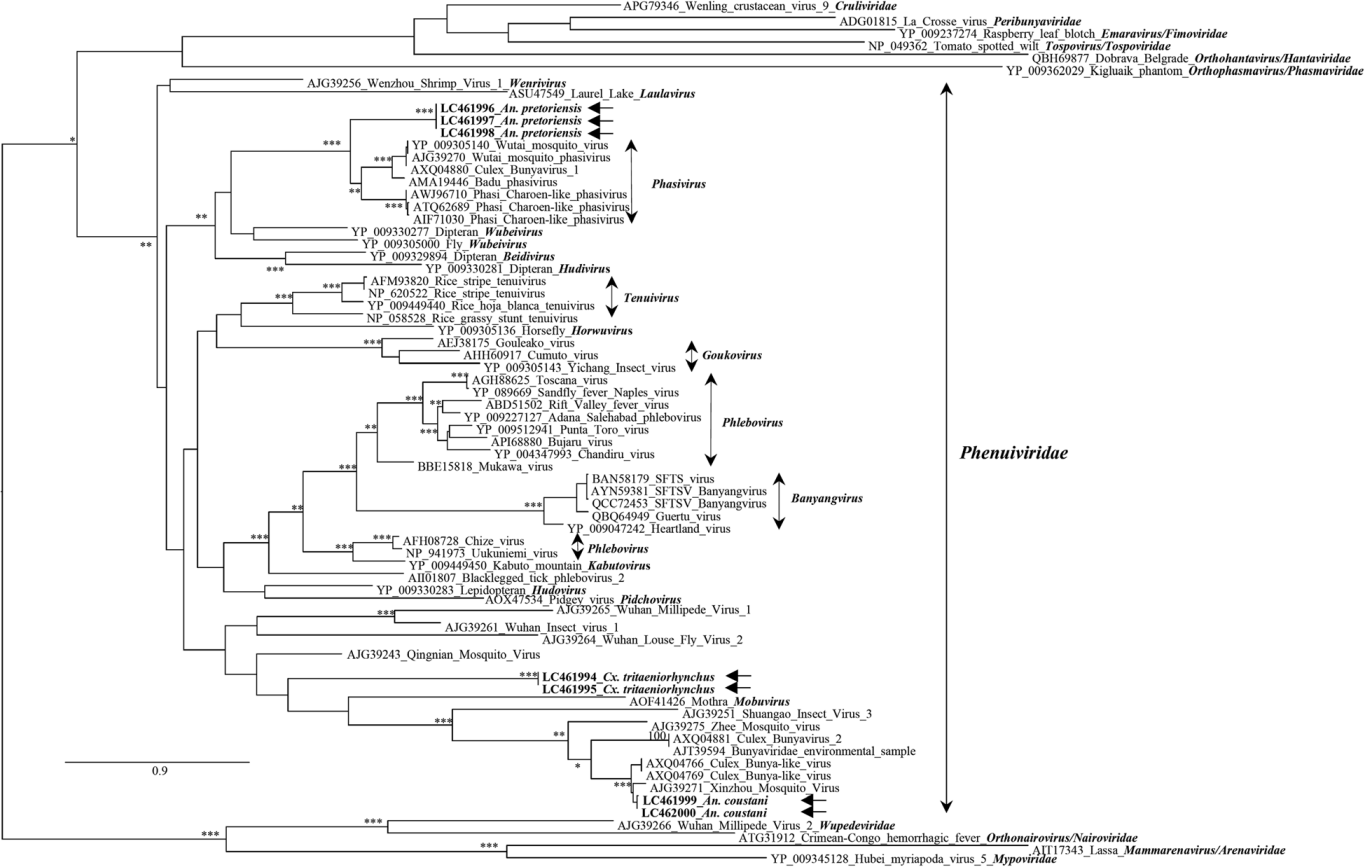
**a** Phylogenetic analysis of partial flavivirus *NS5* nucleotide sequences (0.5 kbp DNA amplicon) from insect-specific flaviviruses. cISF and dISF indicate classical and dual-host associated insect-specific flaviviruses, respectively. At specific branches, the number of “*” indicates the support revealed by the different phylogenetic reconstructions methods used (bootstrap values ≥75% and posterior probability values ≥0.80 were assumed as significant). The sequences described in this work are indicated in bold-face, and their origin (mosquito species) is also indicated. All the sequences used are designated by their respective accession numbers|virus name. The size bar indicates the number of nucleotide substitutions per site. **b** amplification of flavivirus-like from RNA and mosquito genomic DNA using a combination of treatments that included the use/or not (+/) of DNase I followed/or not (+/−) by reverse transcription (RT). The NZYtech ladder VI was used as a molecular mass marker. **c** Structure of the flavivirus wild-type and the NS5 fragments analyzed. The 3′-end of the viral genome is schematically shown at the top. §-Mosquito species could not be confirmed by COI sequence

Curiously, the PCR amplification profiles of the flavivirus RT-PCR reactions frequently revealed (in agarose gels) the presence of an amplicon with approximately 0.5 kbp. This amplicon was observed in association with 31/351 (8.8%) of the pools analyzed, by itself in 9/351 (2.6%) or in combination with the expected 1 kbp DNA fragment in 22/351 (6.3%). However, given its size, it would correspond to a deleted form of the NS5 coding gene suggesting (i) that it might have been amplified from defective viral genomes and/or (ii) rearranged forms of retro-transcribed viral DNA, possibly integrated in mosquito genomes as previously observed [33–35], and/or their resulting transcripts. The association of these smaller sequences with a flavivirus origin was clearly confirmed both by sequence homology searches (using BLASTn) and the reconstruction of phylogenies (Fig. 3a). All six 0.5 kbp amplicons (indicated exclusively by NS5Δ in Fig. 3a) that had been apparently obtained after amplification by RT-PCR from total RNA extracted from mosquito pools were not only clearly part of the cISF radiation but also clustered together in a single, and highly stable monophyletic cluster that subdivides into two subclusters (indicated by *Mansonia*-specific cISF/NS5Δ in Fig. 3a). Moreover, these same 0.5 kbp amplicons could also be obtained when total DNA was used as a template for PCR amplification, and no reverse-transcription had been performed, but when a DNase I treatment preceded reverse-transcription, no 0.5 kbp amplification product was obtained (Fig. 3b). These results show that the origin of the frequently observed 0.5 kbp fragment was not cDNA, but rather corresponded to viral DNA forms (vDNA) contaminating the RNA extracts. Three of these amplicons (indicated by the arrows in Fig. 3a), amplified from mosquito DNA pools of *Ma. africana*, *Ma. uniformis* and *Cx. antennatus*, were cloned and sequenced. Once again, the obtained sequences fell within the same monophyletic cluster. Moreover, when the structure of these DNA fragments was investigated, all of them revealed a similar architecture (Fig. 3c), combining both different sized deletions (down to 1 nt; indicated by Δ) and point mutations.

### Screening of alphaviruses and bunyaviruses, and analysis of phenuivirus L-sequences

Very different results were obtained when either *Alphavirus*-specific primers [36] or those targeting conserved sequences in the RVFV NSs coding-region [37] were used. In fact, neither of these sets of primers revealed the presence of the genomes of these viruses in any of the 351 pools of mosquitoes analyzed. Frequently, the use of the RVFV primers did result in the non-specific amplification of different sized PCR products, many of which were cloned and sequenced. In all cases (results not shown), the obtained sequences confirmed the non-viral origin of these amplicons.

On the other hand, given the overwhelming diversity of the viruses that compose the recently proposed Order *Bunyavirales*, a decision was made not to restrict the screening of bunyaviruses to RVFV, but to extend it, in a smaller subset (43/351) of the pools of mosquitoes collected in different geographic areas of Mozambique, using *Phlebovirus* and *Orthobunyavirus* primers [38, 39]. This subset of 43 pools included the species *Ae. (Adm) fowleri* (*n* = 1), *Ae. (Dic) adersi* (n = 1), *Ae. (Muc) sudanensis* (*n* = 1), *Ae. (Neo) circunluteolus* (*n* = 4), *Ae. (Neo) mcintoshi* (*n* = 2), *Ae. (Ste) aegypti* (*n* = 1), *Ae. (Ste) metallicus* (*n* = 1), *An.(Ano) coustani* (n = 2), *An. (Ano) tenebrosus* (n = 1), *An. (Cel) funestus* (n = 1), *An. (Ano) ziemani* (n = 1), *An. (Cel) pharoensis* (n = 1), *An. (Cel) pretoriensis* (n = 2), *Cq. (Coq) metallica* (n = 1), *Cx. (Cux) antennatus* (*n* = 5), *Cx. Cux) tritaeniorhynchus* (n = 2), *Cx. (Cux) neavei* (n = 2), *Cx. (Cux) pipiens* s.l. (n = 2), *Cx. (Cux) poicilipes* (n = 1)*, Cx. (Cux) zombaensis* (n = 1), *Cx.* sp. (1), *Ma. (Mnd) africana* (*n* = 3), and *Ma. (Mnd) uniformis* (*n* = 4), *Mimomyia (Mim) mimomyiaformis* (n = 1), and one pool of *Ae*. (Neo) sp.

**Fig. 4:**
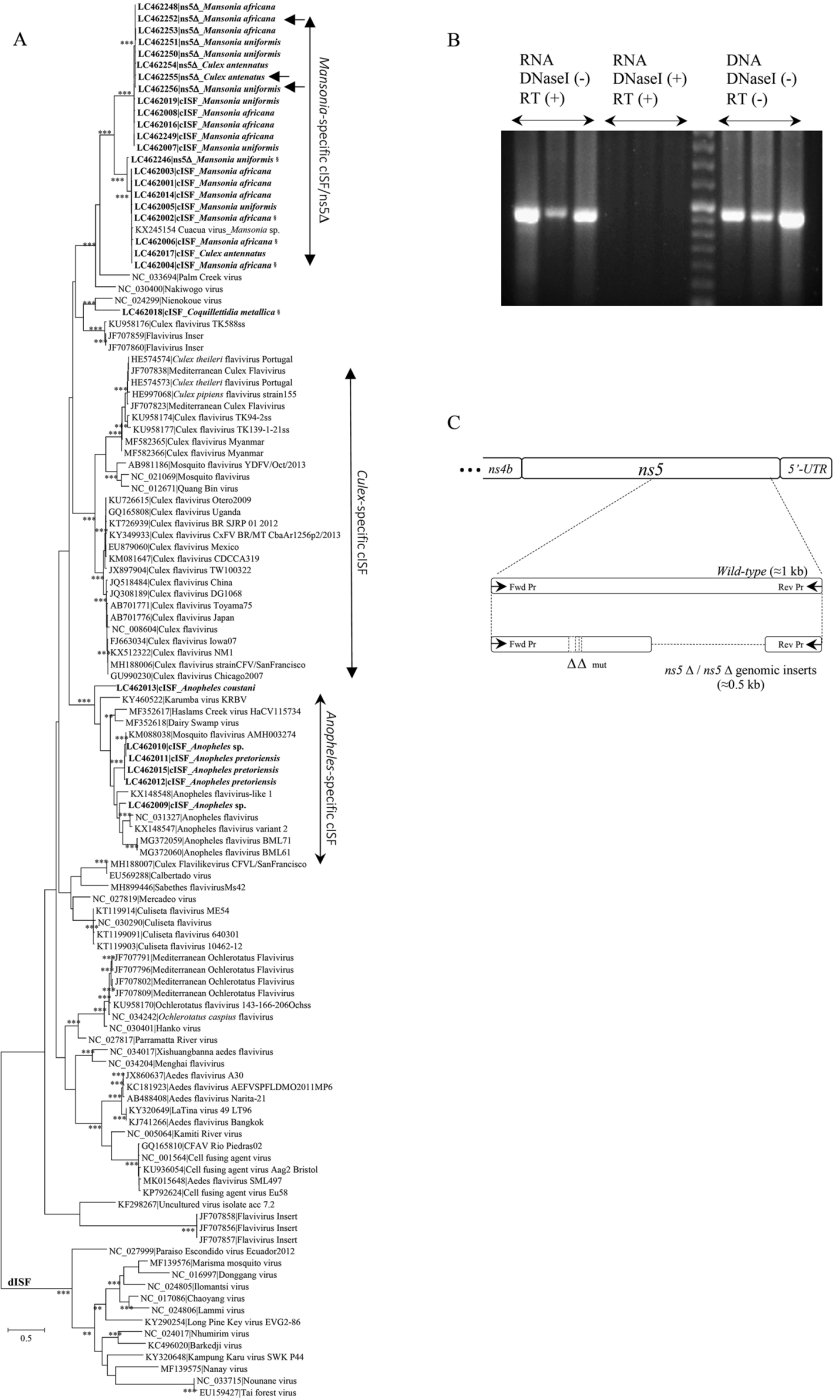
Phylogenetic analysis of partial amino acid sequences of the viral-encoded RNA polymerase of viruses within the Order *Bunyavirales*. At specific branches the number of “*” indicates the support revealed by the different phylogenetic reconstructions methods used, assuming as relevant bootstrap values ≥75% and posterior probability values ≥0.80. The reference sequences used are indicated by their accession number|virus name. The sequences described in this work are indicated by their accession numbers, by the horizontal arrows and in bold-face. The size bar indicates the number of amino acid substitutions per site

Whereas the results that were obtained failed to reveal the presence of *Orthobunyavirus* genomes, in 5 pools, two of *An. coustani* (sequences LC461999, LC462000), two of *An. pretoriensis* (sequences LC461996, LC461997, and LC461998) and one of *Cx. tritaeniorhynchus* (sequences LC 461994 and LC46195) mosquitoes, a DNA fragment with the expected size was, indeed, amplified. All these amplicons were sequenced, but while BLASTn/x sequence searches did indicate a viral origin, unexpectedly they did not seem to have derived from bona fide *Phlebovirus* genomes, and this was confirmed by phylogenetic analysis using an assemblage of *Phlebovirus*, *Bandavirus*, *Banyangvirus,* and *Goukovirus* reference sequences. Regardless of the fact that the *Phlebovirus* group was paraphyletic, the sequences obtained from the analyzed mosquitoes from Mozambique did not cluster in any of the viral *taxa* in the tree, but rather formed 3 independent genetic lineages, as indicated by the arrows in Supplementary Fig. 1. The origin of these viral sequences was investigated using phylogenetic analysis of aligned amino acid sequences of the viral L protein from viruses classified within the different families in the Order *Bunyavirales*. The obtained results (Fig. 4) showed that, while all these sequences were clearly placed within the family *Phenuiviridae*, only two of them clustered with previously known viral references [8, 40, 41], yet in a cluster with no assigned designation. The other five sequences, two amplified from a pool of *Cx. tritaeniorhynchus*, and three others from pools of *An. pretoriensis* and *An. coustani* formed isolated genetic lineages, probably representing new unassigned genera.

### Isolation of viruses using C6/36 cells

In an attempt to isolate some of the identified viruses, six filter-sterilized aliquots of macerates (Moz 39/*Anopheles sp.*, Moz 54/*Cx. tritaeniorhynchus*, Moz 89/*Ma. africana*, Moz 97/*An. coustani*, Moz 98/*An. coustani*, and Moz 160/*An. pretoriensis*) were put in contact with sub-confluent monolayers of C6/36 cells for virus isolation. Viral replication was allowed for 14 days (one blind passage was carried out at the end of the first week), after which the presence of viral genomes was verified by RT-PCR using the same primers used for viral screening. At day three after the first blind-passage, three of these supernatants revealed evident CPE that included an apparent cell growth arrest accompanied by cell rounding, detachment from the flask surface, and sometimes clumping (Supplementary Fig. 2. Surprisingly, none of the primers used revealed the presence of any of the viruses detected during the viral screening. Moreover, as previously the isolation of Negev-like viruses had been associated, in our lab, to CPE similar to the one described above, RT-PCR with a series of primers designed for detection of several subsets of Nelorpiviruses (Carapeta et al., 2015), was performed. Once again, the presence of these viruses was not confirmed. Therefore, to the present day, the identity of the viruses isolated remains to be elucidated, which will be carried out using a metagenomic approach.

## Discussion

In this report, a screening for different groups of RNA viruses targeting the detection of some of those previously shown (genome detection) or suggested (seroprevalence studies) to circulate in Mozambique [23, 29, 42, 43]. This analysis was carried out based on a one-year sampling effort, that amounted to the screening (for viral genomes) of 14,519 mosquitoes from 3 regions of the country. As only female mosquitoes may serve as vectors of viruses to vertebrates, male mosquitoes were excluded from this viral screening. Although the detection of viral agents is facilitated when their presence is associated with visible clinical signs/symptoms in vertebrates, their screening in their natural hosts/vectors may have the advantage of signaling their circulation before any cases of clinical disease, or seroprevalence, are detected. Moreover, a viral screening effort based on the identification of *foci* of disease cases only discloses the circulation of pathogenic viruses, and these have been shown to represent only a part of the virome of mosquitoes [11–14].

The molecular screening that was carried out did not reveal the presence of RVFV or any recognizable pathogenic alphaviruses, bunyaviruses or flaviviruses. These include viruses such as DENV, ZIKV, CHIKV, o’nyong nyong, Sindbis or Middelburg [36]. While the absence of alphaviruses in this viral screening may be intriguing, we must bear in mind that unlike other virus groups (e.g. flaviviruses), alphavirus ISVs have, with exceptions [44, 45], been less frequently reported in viral surveys, while pathogenic alphaviruses such as CHIKV are usually associated with *Aedes* mosquitoes which were clearly underrepresented in our screening. Furthermore, no *Orthobunyavirus* sequences were ever detected in a small sample of the pools analyzed (*n* = 43; the same subset of pools where a survey for *Phlebovirus* genomes was also carried out).

On the contrary, the use of a highly degenerate flavivirus-specific primer set [46] confirmed the presence of multiple genetic lineages of flaviviruses in a large number of pools of mosquitoes. Despite the fact that not all of the obtained amplicons were sequenced, those for which a sequence was obtained were found to segregate in the cISF radiation.

The different genetic lineages of viral *NS5* sequences were apparently associated with multiple species from 4 genera, supporting the perception that cISF are widespread in the natural populations of mosquitoes. Some of these *NS5* sequences seemed to segregate away from previously described viral assemblages and formed isolated branches in phylogenetic trees. Others were joined in clusters with multiple operational taxonomic units that were never exclusively associated with a single species of mosquitoes, adding to the possibility that cISF may not host species-restricted [47, 48]. However, while phylogenetic analysis did suggest a *Culex* origin for one of the sequences (LC462017), given the fact that it was almost identical to many others amplified from *Mansonia* mosquitoes, its association with *Culex* mosquitoes is disputable. Furthermore, although a molecular confirmation of the identity of these mosquitoes was obtained by *COI*-sequence analysis, the sequencing strategy used (Sanger) is a population-approach that only reveals the sequence of the most abundant molecular form in a PCR-product, while minor variants fail to be detected. Therefore, we cannot formally exclude the possibility that sequence LC462017 may have been derived from one/a small number or even body-parts of non-*Culex* mosquitoes (possibly *Mansonia*) originally present in the pool in question.

Surprisingly, in a high number of pools of *Mansonia* spp. (*n* = 29) in one pool of *Anopheles* sp. and another of *Culex* sp. mosquitoes, the flavivirus-specific primers used generated a smaller than expected PCR product, with approximately half the size (≈0.5 kbp). The analysis of some of these smaller amplicons showed that they corresponded to defective versions of the RdRp coding sequence and their origin was found to be DNA (vDNA), rather than RNA. For all those cases where a nucleotide sequence could be obtained, a shared ancestry between the latter and bona fide viral *NS5* sequences (obtained by RT-PCR) was also revealed.

While we cannot ascertain, at this stage, whether the flavivirus vDNA forms are present as part of the host genome (endogenized), or whether they exist in the form of a stable extra-chromosomal DNA element, flavivirus-like sequences have been known to occur in the genome of mosquitoes for over a decade, especially in association with *Aedes* mosquitoes [33, 34]. While the sequence of a vDNA amplicon amplified from *Anopheles* could not be obtained due to technical difficulties, the fact that virtually identical vDNA sequences could be amplified from DNA extracts of *Mansonia* and *Culex* mosquitoes is hard to explain given the evolutionary divergence of these *taxa*. Moreover, while these vDNA forms could result from exposure of these mosquitoes to a common source of viruses, the possibility of a contamination of pools of *Culex* mosquitoes with even a limited amount of the highly abundant *Mansonia* specimens, cannot be discarded.

Whereas the presence of bacterial symbionts of mosquitoes can alter the competence of mosquitoes for transmission of pathogenic viruses [49], to what extent the same applies to the persistent presence of insect-specific viruses in insect cells is still open to discussion. However, the highly rearranged *NS5* sequences found in this study seem to exclude the possibility that translation of an RNA transcribed from them might result in an active protein. In any case, they could participate in the establishment of persistence viral infections by controlling the siRNA response, as previously suggested [50].

Given the a priori specificity of the primers used for the screening of *Phlebovirus* sequences, the observation of a specific amplicon in association with 5 pools of 2 different species of *Anopheles* (*An. coustani* and *An. pretoriensis*) and one species of *Culex* (*Cx. tritaeniorhynchus*) mosquitoes suggested that these viruses might have been detected. However, the different phylogenetic analyses were congruent in showing (i) their inclusion in the *Phenuiviridae* family, (ii) but their exclusion from the *Phlebovirus* genus, (iii) and their separation into three genetic lineages. Two of these sequences did segregate in a stable monophyletic cluster defining a genetic lineage with no assigned designation, but that included sequences previously detected in other studies [8, 40, 41], while the other five formed two genetic lineages with no associated references.

Although no recognizable pathogenic viruses were identified in the course of this work, this may result from a combination of multiple factors that include sampling bias. In fact, collections did not focus on settings where DENV/ZIKV/CHIKV were previously known to circulate in Mozambique [23, 42, 43], but rather on areas where RVFV had been detected [18, 19]. On the contrary, *Mansonia* and *Culex* mosquitoes clearly dominate the collections in the 3 provinces of Mozambique that were the focus of this study. However, pathogenic flavivirus such as the Spondweni virus (the closest known relative to ZIKV), have indeed been isolated from *Ma. africana* and *Ma. uniformis* [11], as well as from *Culex quinquefasciatus* mosquitoes in Haiti [51]. Association of other pathogenic flaviviruses with *Mansonia* sp. mosquitoes include the S. Louis encephalitis and West-Nile viruses (which also use *Culex* sp. for their natural maintenance), alphaviruses (including Venezuelan equine encephalitis virus), orthobunyaviruses [52], and phleboviruses, including RVFV [11].

While sampling bias may partially explain the absence of some of the arboviruses that have been previously shown to circulate in Mozambique, other factors may also explain the results obtained. These include a low natural incidence of arboviruses in the areas where mosquitoes were collected, or the concurrent absence of recorded cases of human/animal disease cases associated to the circulation of viruses such as RVFV during the mosquito collection periods. Furthermore, a technical limitation of this study is associated with the use of a less technologically advanced virus detection approach based on convectional RT-PCR, as opposed to addressing viral screening with a bona fide metagenomic experimental design combined with the use of NGS sequencing methods. To the best of our knowledge, this study and the previous detection of ISF in *Mansonia spp* [29], are the only recent virus surveys using mosquitoes from Mozambique, and clearly demonstrates the dire need for such surveys that might clarify their epidemiology.

The attempted isolation of some of the viruses identified in this work in insect cells was not successful. This fact may probably result from a combination of factors that include the use of only one blind passage and a single cell-line. Indeed, while C6/36 cells have been extensively used for the isolation of ISVs they may not be susceptible and well as permissive to all insect viruses. In this regard, it should be added that some ISF seem to be restricted to theirs hosts [53], and this may indicate that use of C6/36 cells, although convenient, may not have been ideal. For more clarification further analysis involving cell culture attempts using a larger number of cell lines originating from different species of mosquitoes is recommended. While a very short blind-passage history may have compromised the production of a high titer viral suspension, in truth the exact same RT-PCR protocols were used to screen the presence of viral genomes in mosquito macerates and culture supernatants. Moreover, only after a single blind-passage, 50% of the cultures did evidence unambiguous CPE. Taking into account the protocol used, this CPE most probably was due to viral replication.

The nature of these viruses is currently under investigation. Furthermore, bioinformatics investigations for producing the unidentified CPE observed in inoculated cells is encouraged for better understanding the prevalence of insect-specific viruses in many genera of mosquitoes [54, 55]. While these efforts should be ideally addressed using unbiased and high-throughput experimental approaches (metagenomics/NGS), the direct screening of other frequently found ISVs, including alphamesoniviruses [56] could also be pursued using a direct targeting strategy with *taxon*-specific primers.

## Conclusion

This study reports for the first-time novel insect-specific flaviviruses and phenuiviruses, as well as frequent flavivirus-like viral DNA forms in several widely known vector species. While a large diversity of ISVs have been found on a global scale [57, 58] in association with a plethora of insect hosts, this work extends the results of the sole study that had, up to the present day, revealed their presence in Mozambique [29]. Although this survey did not disclose the circulation of pathogenic arboviruses, it confirmed the circulation of different RNA viruses that are present in mosquitoes from Mozambique. This article represents our professional endeavor to help to elucidate and provide higher resolution information on arboviruses vectors hotspot, transmission dynamics and routes in Mozambique and is of utmost importance to inform the establishment of a vector control program for arbovirus in the country and other region sharing the same pattern.

## Methods

### Study area and mosquito collection

A total of 14,519 mosquitoes were collected in rural settings in Mozambique (located in west southern Africa) between November 2014 and December 2015 as part of the work of Abílio, AP (In Preparation) at Massingir (in the province of Gaza), Namaacha (in the province of Maputo), and Mopeia (in the province of Zambézia) (Fig. 1). The general biotypes for Goba were savanna with medium grassland located around 10 to 500 m from a water stream. Collection sites in Massingir and Mopeia corresponded to forest environments located closed to the Lipompo and Zambezi rivers, respectively. The mosquitoes were collected using a combination of sampling methods that included indoor resting, tent collections and those carried out using CO_2_-baited miniature CDC-light traps. These mosquitoes were stored in dry ice, and then transported to the laboratory for sorting and taxonomic identification using keys proposed by Gillies and Coetzee [59] and Jupp [60]. The manipulations of specimens for identification were carried out at temperatures close approximate to 0 °C under a stereomicroscope equipped with an ice block. Male and blood-fed specimens were excluded from this study. All samples were then stored at − 80 °C until viral screening was carried out.

### Preparation of mosquito homogenates, and nucleic acid extraction

The preparation of mosquito homogenates was based on a preliminary grouping of the collected and identified specimens in pools according to their species, sex, geographic origin, and blood-fed status. These mosquitoes were mechanically disrupted in 15 ml Falcon tubes by vortexing using glass-beads and aluminum oxide in 1 ml of phosphate buffer saline (PBS) buffer. After 3 pulses of 1 min (with 30 s breaks on ice), the mosquito macerates were clarified by centrifugation, as previously described (Carapeta et al., 2015). RNA, as well as DNA, were extracted from 200 μl of clarified mosquito homogenate using NZYol® (NZYTech, Portugal), as indicated by the supplier. The extracted RNA was dissolved in 30 μl nuclease-free water, while the obtained DNA sediments were dissolved in 40–100 μl using a 1:1 mixture of 8 mM NaOH and TE buffer (Tris 100 mM, EDTA 1 mM, pH = 7).

### Viral genome detection

The extracts of total RNA served as a template for the synthesis of cDNA, that was carried out with the NZY First-Strand cDNA Synthesis Kit (NZYTech, Portugal) using random hexamers, and a thermal profile including 10 min at 25 °C, 45 min at 52 °C and 10 min at 80 °C (for enzyme inactivation), followed by treatment with RNaseH (20 min at 37 °C).

Detection of flavivirus *NS5* sequences (encoding the viral RNA-dependent RNA polymerase, or RdRp) was carried out using previously described primers and reaction conditions [46]. A generic PCR method using degenerate primers targeting the *nsP4* gene (also encoding the viral RdRp) was used to detect the presence of the genomes of alphaviruses [36], while RVFV genomic NSs coding sequences were tentatively detected as previously described [61]. Finally, the presence of phleboviruses and orthobunyaviruses L sequences (also encoding an RdRp) was investigated using the ppL1/ppL2 sets of primers/reaction conditions previously described by Matsuno and others [38] and the technical modifications suggested by Pereira and others [62], or as defined elsewhere [39]. Nelorpivirus detection was carried out as previously defined [63]. All the PCR primers and thermal profiles used are listed in Supplementary Table 1. PCR amplifications were carried out using NZYTaq 2X Green Master Mix (NZYTech, Portugal). The obtained amplicons were purified and directly sequenced or cloned in either pGEMT-easy® (Promega, USA) or pNZY28-A using the NZY-A PCR cloning kit (NZYTech, Portugal), followed by DNA sequencing of individually purified plasmid recombinant-DNA molecules.

### Cell culture and virus isolation

*Aedes (Ste) albopictus* C6/36 cell line was used for virus isolation. Cells were maintained at 28 °C (in the absence of CO_2_) in L-15 Leibovitz Medium (Lonza, USA) supplemented with 10% heat-inactivated fetal bovine serum (FBS) (Lonza, USA), 2 mM L-glutamine (Gibco BRL, USA), 100 U/ml penicillin and 100 μg/ml streptomycin (Gibco BRL, USA) and 1 × tryptose phosphate broth (AppliChem GmbH, Germany). Approximately 500 μl of filter-sterilized mosquito homogenate were diluted in the same volume of phosphate buffered saline (PBS), and inoculated onto semi-confluent layers of C6/36 cells grown in T25 culture flasks (Nunc, Denmark). After 1 h at room temperature (for viral adsorption), the viral inoculum was removed, 5 ml of L-15 Leibovitz Medium (2% FBS) was added to each flask, and the cell cultures were incubated at 28 °C for a week. Culture supernatants collected after a single blind-passage were used as viral stocks and stored at − 80 °C. Cytopathic effect (CPE) was determined by microscopic observation of the inoculated cell cultures.

### DNA sequencing and genetic analyses

Multiple alignments of nucleotide (nt) or amino acid (aa) sequences were performed using the iterative G-INS-I and E-INS-I methods as implemented in MAFFT vs. 7 [64] followed by editing using both GBlocks [65], and visual inspection. The multiple sequence alignments of nucleotide sequences were systematically verified to ensure the correct alignment of homologous codons using BioEdit 7.0.5 [66].

Phylogenetic trees were constructed using both Maximum Likelihood (ML) and Bayesian approaches. The best-fitting evolutionary models used were those suggested by JModeltest2 (Darriba et al., 2012) and W-IQ-tree (Trifinopoulos et al., 2016) for the analysis of nt (GTR + Γ + I: GTR-General Time Reversal, Γ-Gamma distribution, I-proportion of invariant sites) or aa alignments (LG + Γ: Le-Gascuel, Γ-Gamma distribution). Phylogenetic analyses based on the ML optimization criterion were carried out using the Mega 6.0 software [67], and the stability of the obtained tree topologies assessed by bootstrapping with different re-samplings of the original aligned positions (1000 for nt alignments, 100 for aa sequence data). Phylogenetic reconstructions following a Bayesian approach were carried out by running two independent Markov chain Monte-Carlo (MCMC) analyses using BEASTv1.7.5 [68], assuming a relaxed uncorrelated lognormal molecular clock model [69] as suggested by the ML Clock Test implemented in Mega 6.0. The MCMC chains were run until 100,000,000 states were sampled using both logistic population growth and Gaussian Markov random field/GMRF skygrid demographic priors. The Tracer software (http://beast.bio.ed.ac.uk/tracer) was used to diagnose stationarity and adequate (> 300) effective sample size (ESS). The trees were logged on every 5000th MCMC step, and the tree sample was summarized using TreeAnnotator v1.8.3 as maximum clade credibility (MCC) trees, with median heights used as the node heights in the tree, after discarding 10% of them as burn-in. The FigTree v1.4.2 software was used to visualize the phylogenetic trees (http://tree.bio.ed.ac.uk/software/figtree/).

The molecular confirmation of the morphological identifications of mosquitoes was carried out on the basis of the analysis of the barcoding section (from positions 58 to 705 encoding the N-terminal section of the mitochondrial cytochrome oxidase subunit I - COI) essentially using Bold Systems-v4 (available at http://www.boldsystems.org/).

The nt sequences obtained in the course of this study were deposited in the GenBank/ENA/DDBJ databases under accession numbers LC461994-LC462019, and LC-462246-LC462257, and LC517270-LC517293. The reference sequences used for analyses presented in this manuscript where directly downloaded from the public sequence databases. Whenever necessary, nt sequence similarity searches were carried out using BLASTn, and BLASTx (https://blast.ncbi.nlm.nih.gov/Blast.cgi).

## Supplementary information

**Additional file 1: Supplementary Figure 1.** Microscopic observation of C6/36 cells mock-infected cells (A; 300×), or after infection (day 3) with viruses present in three independent pools of *Ma. uniformis*, *An. ziemani*, and *An. pretoriensis* mosquitoes collected in Mozambique.

**Additional file 2.**

**Additional file 3: Supplementary Table 1.** PCR primers and thermal profiles used in this work.

## Data Availability

The datasets used and/or analyzed during the current study are available from corresponding author on reasonable request.

## Abbreviations

DNA: Deoxyribonucleic acid
RNA: Ribonucleic acid
cDNA: complementary DNA
PCR: Polymerase chain reaction
RT-PCR: Reverse transcriptase-PCR
