# Supplementary figures and images for: A survey of RNA viruses in mosquitoes from Mozambique reveals novel genetic lineages of flaviviruses and phenuiviruses, as well as frequent flavivirus-like viral DNA forms in Mansonia
Source: BMC Microbiol. 2020 Jul 28;20:225. doi: 10.1186/s12866-020-01905-5 (PMC7385898; doi:10.1186/s12866-020-01905-5)

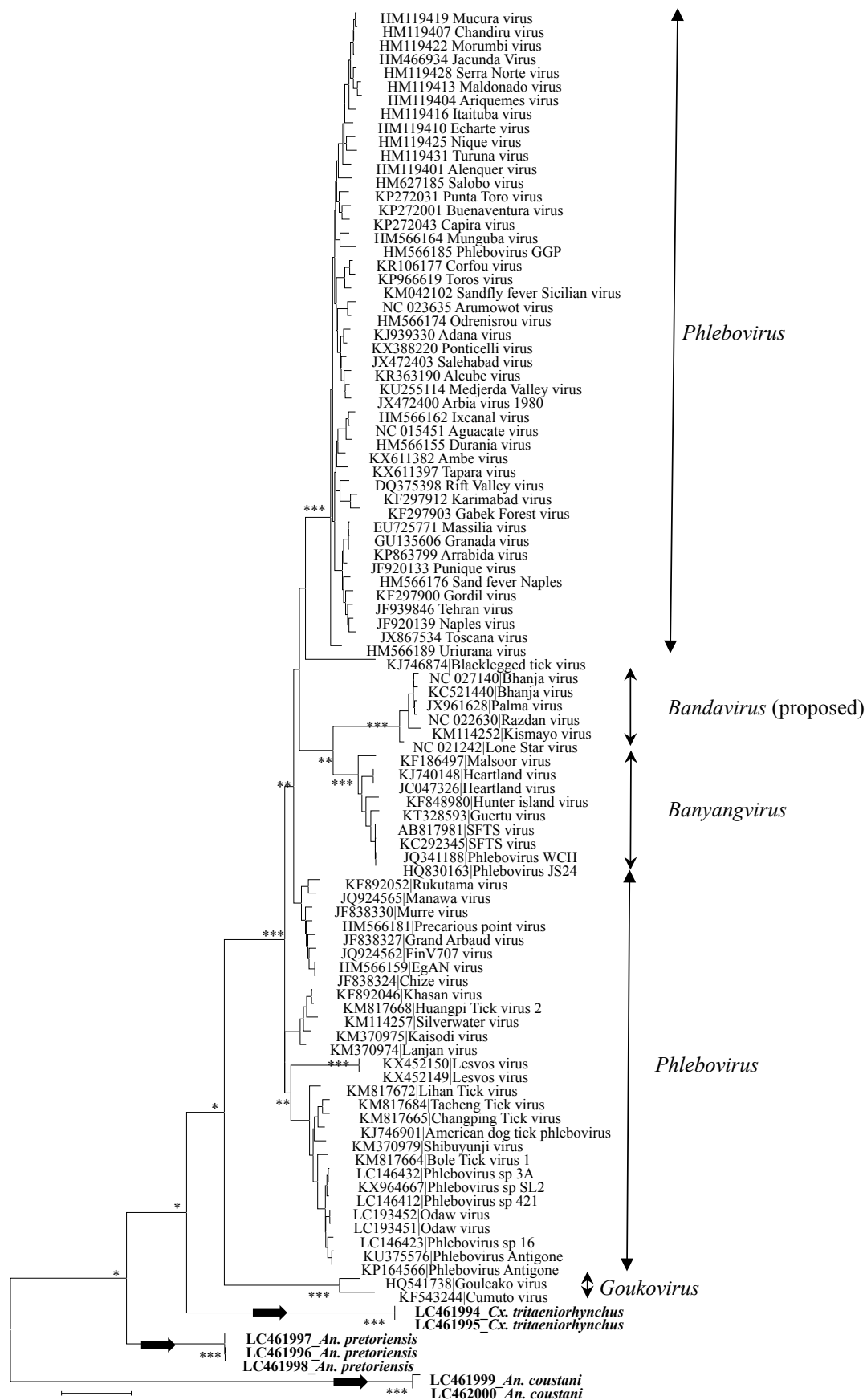

Supplement: Supplementary file 1 — Additional file 1: Supplementary Figure 1. Microscopic observation of C6/36 cells mock-infected cells (A; 300×), or after infection (day 3) with viruses present in three independent pools of Ma. uniformis, An. ziemani, and An. pretoriensis mosquitoes collected in Mozambique. [file 12866_2020_1905_MOESM1_ESM.pdf]

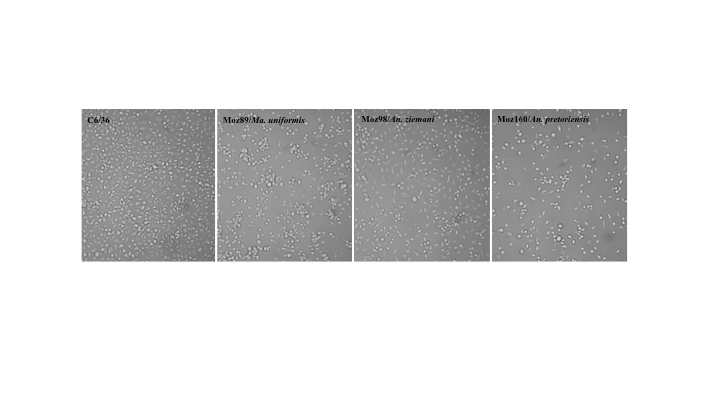

Supplement: Supplementary file 2 — Additional file 2. [file 12866_2020_1905_MOESM2_ESM.tiff]
