# Supplementary material for: A survey of RNA viruses in mosquitoes from Mozambique reveals novel genetic lineages of flaviviruses and phenuiviruses, as well as frequent flavivirus-like viral DNA forms in Mansonia
Source: BMC Microbiol. 2020 Jul 28;20:225. doi: 10.1186/s12866-020-01905-5 (PMC7385898; doi:10.1186/s12866-020-01905-5)
Supplement: Supplementary file 3 — Additional file 3: Supplementary Table 1. PCR primers and thermal profiles used in this work. [file 12866_2020_1905_MOESM3_ESM.docx]

Supplementary Table 1 - PCR primers and thermal profiles used in this work.

| **Target gene (virus type)** | **Primer sequence (5’-3’)** | **Thermocycling conditions** | | | | **Reference** |
| --- | --- | --- | --- | --- | --- | --- |
| *ns5* (Flaviviruses) | 1^st^ PCR | 95 ºC - 5 min; 45 cycles [95 ºC - 1 min; 50 ºC - 4 min; 72 ºC - 1 min]; 72 ºC - 5 min | | | | Vásquez et al., 2012 |
|  | 1NS5F: GCATCTAYAWCAYNATGGG |  |  |  |  |  |
|  | 1NS5R: CCANACNYNRTTCCANAC |  |  |  |  |  |
|  | 2^nd^ PCR | Same as above | | | |  |
|  | 2NS5F: GCNATNTGGTWYATGTGG |  |  |  |  |  |
|  | 2NS5R: CATRTCTTCNGTCGTCATCC |  |  |  |  |  |
|  |  |  | | | |  |
| *nsP4* (Alphaviviruses) | 1^st^ PCR | 94 ºC - 2 min; 45 cycles [94 ºC - 30 sec; 52 ºC - 1 min; 72 ºC - 30 sec]; 72 ºC - 5 min | | | | Sánchez-Seco et al., 2001 |
|  | Alpha1+: GAYGCITAYYTIGAYATGGTIGAIGG |  |  |  |  |  |
|  | Alpha1-: KYTCYTCIGTRTGYTTIGTICCIGG |  |  |  |  |  |
|  | 2^nd^ PCR | Same as above | | | |  |
|  | Alpha2+: GIAAYTGYAAYGTIACICARATG |  |  |  |  |  |
|  | Alpha2-: GCRAAIARIGCIGCIGCYTYIGGICC |  |  |  |  |  |
|  |  |  | | | |  |
| *NSs* (RVFV) | 1^st^ PCR | 95 ºC - 1 min; 45 cycles [95 ºC - 30 sec; 55 ºC - 1 min; 72 ºC - 1 min]; 72 ºC - 5 min | | | | Sall et al. et al., 2001 |
|  | NSca: CCTTAACCTCTAATCAAC |  |  |  |  |  |
|  | NSng: KYTCYTCIGTRTGYTTIGTICCIGG |  |  |  |  |  |
|  | 2^nd^ PCR | Same as above | | | |  |
|  | NS3a: ATGCTGGGAAGTGATGAGCG |  |  |  |  |  |
|  | NS2g: GATTTGCAGAGTGGTCGTC |  |  |  |  |  |
|  |  |  | | | |  |
| *ORF1* (Negev-like viruses) | 1^st^ PCR  NegeF: CAYGTRAARATYTTCTGCGAYATGTC  NegevinR: TAATCGTTTGTGCGGTARACATTGAGGC | 95 ºC - 2 min; 45 cycles [95 ºC - 30 sec; 55 ºC - 30 sec; 72 ºC – 1 min]; 72 ºC - 5 min | | | | Carapeta et al., 2015 |
|  | 2^nd^ PCR |  | | | |  |
|  | NegevinF: AGTGCTTCAACGTGACATTCCCCCGTCC  NegevinR: TAATCGTTTGTGCGGTARACATTGAGGC | Same as above | | | |  |
|  |  |  | | | |  |
| *ORF1* (Loreto-like viruses) | \| 1^st^ PCR  LorF: CGGCAATTTGGAATCGAAGAGGAACTTGTC  LorRout: CCACATGAAGGAGGAAGTGTACAACC \| \| --- \| \| 2^nd^ PCR  LorF: CGGCAATTTGGAATCGAAGAGGAACTTGTC \| \| LorR: TGTGCGATGAACTTCGATACATTCCGGGTC \| | 95 ºC - 2 min; 45 cycles [95 ºC - 30 sec; 55 ºC - 30 sec; 72 ºC – 1 min]; 72 ºC - 5 min  Same as above | | | | Carapeta et al., 2015 |
|  |  |  | | | |  |
| *ORF1* (Denzidougou-like viruses) | \| 1^st^ PCR  DenzF: TAATTTGTGYGTTACYGCTCTKACTMGGCACAC  DenzR: ATACGAACYTTRGGATTRCGTTTCAGAGAC \| \| --- \| \| 2^nd^ PCR  DenzF: TAATTTGTGYGTTACYGCTCTKACTMGGCACAC \| \| DenzinR: GCKGGAGCAGGAGTGCTCAACMMCGG \| | 95 ºC - 2 min; 45 cycles [95 ºC - 30 sec; 55 ºC - 30 sec; 72 ºC – 1 min]; 72 ºC - 5 min  Same as above | | | | Carapeta et al., 2015 |
| *L* (Phleboviruses) | TBPVL2759F: CAGCATGGIGGICTIAGAGAGAT  TBPVL3267R: TGIAGIATSCCYTGCATCAT  HRT-GL2759F: CAGCATGGIGGIYTIAGRGAAATYTATGT | 95 ºC - 2 min; 45 cycles [95 ºC - 30 sec; 55 ºC - 30 sec; 72 ºC - 30 sec]; 72 ºC - 5 min | | | | Matsuno et al., 2015 |
|  | HRT-GL3276R: GAWGTRWARTGCAGGATICCYTGCATCAT |  |  |  |  |  |
|  |  |  |  | |  |  |
| *L* (Orthobunyaviruses) | 1^st^ PCR  OrthoF12: TRACTGARCCWTCTMGATATATGATAATGAAYT | 95 ºC - 2 min; 45 cycles [95 ºC - 30 sec; 53 ºC - 45 sec; 72 ºC – 45 sec]; 72 ºC - 5 min | | | | Silva et al. (2019) |
|  | OrthoR1: CATCTTGDGCACTCCATTTTGACATRTCHGC |  |  |  |  |  |
|  | 2^nd^ PCR  OrthoF12: TRACTGARCCWTCTMGATATATGATAATGAAYT | Same as above | |  |  |  |
|  | OrthoR2: CATACATRCACATYTTDGCTTCAAATTC |  |  | |  |  |
|  |  |  | | | |  |
| *COI* | LCO1490: ggtcaacaaatcataaagatattgg  HC02198:taaacttcagggtgaccaaaaaatca | 95 ºC - 5 min; 40 cycles [95 ºC - 30 sec; 48 ºC – 30 sec; 72 ºC – 45 sec]; 72 ºC - 5 min | | | | Folmer et al., 1994, Cook et al., 2009 |
|  |  |  | | | |  |

I-inosine, K-G or T, M-A or C, N- any base, R-A or G, S-C or G, W-A or T, Y-C or T.
